# 克里唑替尼治疗晚期非小细胞肺癌的临床研究进展

**DOI:** 10.3779/j.issn.1009-3419.2013.06.09

**Published:** 2013-06-20

**Authors:** 海波 朱, 小玉 徐, 玲 王

**Affiliations:** 1 116044 大连，大连医科大学 Dalian Medical University, Dalian 116044, China; 2 116011 大连，大连医科大学附属医院肿瘤科 Department of Oncology, First Affiliated Hospital of Dalian Medical University, Dalian 116011, China

**Keywords:** ]克里唑替尼, 肺肿瘤, EML4-ALK, ROS1, Crizotinib, Lung neoplasms, EML4-ALK, ROS1

## Abstract

目前在非小细胞肺癌（non-small cell lung cancer, NSCLC）的治疗中，靶向药物治疗占有举足轻重的地位。继表皮生长因子受体酪氨酸激酶抑制剂（epidermal growth factor receptor tyrosine kinase inhibitor, EGFR-TKI）之后，针对棘皮动物微管相关蛋白4-间变性淋巴瘤激酶（echinoderm microtubule associated protein like 4-anaplastic lymphoma kinase, *EML4-ALK*）融合基因突变为靶点的克里唑替尼（crizotinib）成为了NSCLC靶向治疗领域的焦点。Ⅰ期、Ⅱ期临床试验均已证实：crizotinib治疗EML4-ALK阳性晚期NSCLC患者有效，并能改善患者症状，毒副作用小，患者耐受性较好。近期发现crizotinib对ROS1受体酪氨酸激酶也具有抑制作用。Crizotinib在*ROS1*基因重排NSCLC中显示出了非常明显的抗肿瘤活性。与其它TKIs一样，crizotinib也存在耐药现象，其耐药机制待进一步研究。现就crizotinib作用机制、药代动力学及治疗晚期NSCLC的临床研究进展做一综述。

## Abstract (en)

At present, in the treatment of non-small cell lung cancer (NSCLC), targeted therapy has an important status. After epidermal growth factor receptor tyrosine kinase inhibitor (EGFR-TKIs), crizotinib targeted at *EML4-ALK* fusion gene becomes a significant drug of molecular targeted therapy in NSCLC. Phase Ⅰ and Ⅱ clinical trials prove that crizotinib is effective for treatment of activating *EML4-ALK* mutation in advanced NSCLC patients, little side-effect, and well tolerated. Recently, crizotinib can inhibit ROS1 receptor tyrosine kinase and show extraordinary significant antitumor activity in ROS1-rearranged NSCLC. Drug resistance also exists in crizotinib. The mechanism of drug resistance needs further research. In this study, a review is performed in the mechanism and pharmacokinetics of crizotinib, and the clinical progress of treatment in advanced NSCLC.

肺癌是全世界最常见的恶性肿瘤之一，也是癌症死亡的主要原因^[1]^。肺癌预后差，中位生存期不到1年，肺癌患者的5年生存率仅16%。传统化疗对晚期非小细胞肺癌（non-small cell lung cancer, NSCLC）患者的疗效已进入平台期^[2]^，近年来以（epidermal growth factor receptor, EGFR）突变为靶点的靶向治疗药物（包括吉非替尼与厄洛替尼）对特定基因亚型的NSCLC患者有明显的治疗效果^[3, 4]^。*EML4-ALK*^[5]^、*ROS1*^[6]^是新发现的NSCLC驱动基因，crizotinib是EML4-ALK、间叶细胞表皮生长因子（mesenchymal epithelial growth factor, c-Met）、肝细胞生长因子受体（hepatocyte growth factor receptor, HGFR）激酶的选择性抑制剂^[7]^，已在临床研究中取得了较好的疗效。

## 1 作用机制

Crizotinib是一种口服的ALK与c-Met酪氨酸激酶ATP竞争性抑制剂^[7]^。分子式：C_21_H_22_Cl_2_FN_5_O。化学式：(R)-3-[1-(2, 6-dichloro-3-fluorophenyl)ethoxy]-5-[1-(piperidin-4-yl)-1H-pyrazol-4-yl]pyridin-2-amine^[8]^。在NSCLC中，染色体2p上的染色体倒置形成了异常的*EML4-ALK*融合基因。因此，ALK酪氨酸激酶通过异常活化下游磷脂酰肌醇3激酶一蛋白激酶B信号传导通路，引起肿瘤细胞生长、增殖、抗凋亡^[9-11]^。当ALK激酶活性被抑制，能从EML4-ALK NSCLC细胞系和小鼠模型中观察到肿瘤细胞凋亡以及肿瘤收缩^[12]^。Crizotinib通过剂量依赖的方式抑制ALK与c-Met激酶磷酸化，从而抑制细胞增殖^[7, 13]^。

## 2 药代动力学

一项由167例受试者参与的Ⅰ期临床研究对crizotinib药代动力学进行了评价^[14]^。结果显示：单次口服给药，血药浓度达峰值中位时间为4 h-6 h。250 mg，2次/d，血药浓度15 d内达稳态，中位血清蓄积率为4.8，中位半衰期为42 h。单次口服250 mg剂量后，crizotinib的平均绝对生物利用度为43%（范围：32%-66%）。年龄、性别、种族或体重对crizotinib的药代动力学无明显影响^[14]^。Crizotinib在ALK阳性NSCLC患者与其它类型肿瘤患者体内的药动学参数相似；在亚裔患者体内的C_max_和AUC平均值分别为非亚裔患者的1.57倍和1.50倍^[14]^。

## 3 Crizotinib治疗EML4-ALK阳性NSCLC患者的临床试验

Crizotinib是2005年合成的一种口服的Met和ALK强效抑制剂。2007年Soda等^[5]^在1例NSCLC患者的组织标本中首次发现*EML4-ALK*融合基因。此后研究^[15]^发现，NSCLC患者中有3%-7%的患者含有该融合基因。2008年首次在EML4-ALK阳性的肿瘤患者中观察到crizotinib的临床疗效。Crizotinib治疗EML4-ALK阳性NSCLC患者的Ⅰ期、Ⅱ期和Ⅲ期临床试验正在世界各国展开。基于一项Ⅰ期临床试验^[16]^和一项Ⅱ期临床试验^[17]^的试验数据，crizotinib于2011年8月26日获美国FDA批准，用于治疗ALK阳性的局部晚期或转移的NSCLC。

### 3.1 Ⅰ期临床试验

Kwak等^[7]^进行的一项Ⅰ期临床试验，共入组82例*EML4-ALK*融合基因阳性的NSCLC患者，受试者口服crizotinib（250 mg, bid）。经6.4个月的治疗后，客观有效率（objective response rate, ORR）为57%，1例完全缓解，46例部分缓解，27例（33%）患者疾病稳定。在该数据发表前仍有63例还在接受crizotinib治疗，预计无进展生存期（progression free survival, PFS）为72%，中位PFS尚未统计。2011年ASCO会议上，Camidge教授^[16]^公布了一项研究结果。该研究是由119例局部晚期或转移性的EML4-ALK阳性NSCLC患者参加的多中心、单组Ⅰ期临床试验。中位年龄51岁（21岁-79岁），57%患者不吸烟，97%为腺癌。口服crizotinib（250 mg, bid），其ORR为61%，PFS达10个月，治疗的中位有效时间为48.1周，第8周和第16周疾病控制率分别为79%和67%，该研究数据仍在更新。与NSCLC二线化疗药物仅10%有效率相比，crizotinib在EML4-ALK阳性患者中的高有效率令人鼓舞。

同年ASCO会议上Shaw等^[18]^在既往Ⅰ期临床研究的基础上，选取以下3组样本数据进行分析。EML4-ALK阳性实验组：82例EML4-ALK阳性且接受crizotinib治疗的患者；EML4-ALK阳性对照组：37例EML4-ALK阳性未接受crizotinib治疗的患者；EML4-ALK阴性对照组：253例EML4-ALK阴性患者。所有受试者均为晚期NSCLC患者。研究结果示：EML4-ALK阳性实验组患者1年和2年总生存率（overall survival, OS）分别为77%和64%，中位OS尚未统计。EML4-ALK阳性对照组患者1年和2年OS分别为73%和33%，中位OS为20个月。EML4-ALK阳性对照组中受试者均为非韩国裔，因此Shaw等选取EML4-ALK阳性实验组中的56例非韩国裔与其进行亚组分析。这两组人群构成相似：年龄（中位年龄：51岁*vs* 51岁，*P*=0.97）、性别（女性比例：57% *vs* 46%，*P*=0.4）、吸烟史（不吸烟者：68% *vs* 79%，*P*=0.33）、任何时间出现脑转移（49% *vs* 48%，*P*=1.00）、入组前数（平均数：2.05 *vs* 2.09，*P*=0.17）和入组前化疗的种类。EML4-ALK阳性实验组32例在二、三线应用crizotinib的患者的生存情况明显优于相应的EML4-ALK阳性对照组24例二线化疗的患者（*P*=0.004）: 1年生存率71% *vs* 46%，2年生存率61% *vs* 9%，而中位生存时间EML4-ALK阳性实验组还未统计，EML4-ALK阳性对照组为11个月。在二线使用crizotinib的123例EML4-ALK阴性患者中，1年生存率和2年生存率分别为49%和33%，中位生存时间为11个月。虽然该项研究未能统计出EML4-ALK阳性crizotinib治疗组患者的中位OS，但是可以看出，crizotinib对EML4-ALK阳性的NSCLC患者的总生存率有明显改善。

### 3.2 Ⅱ期临床试验

一项多中心、单臂的Ⅱ期临床试验（PROFILE1005）^[19]^正在进行。入组的是经FISH证实为EML4-ALK阳性的NSCLC患者，预计入组400例患者。这些患者口服crizotinib 250 mg，每日2次，21 d为1个周期。在2011年ASCO会议上，Crinò等^[17]^公布了这项临床试验的初步结果：136例患者进行了药物安全性评估，109例患者进行了临床结局评价，76例患者进行了肿瘤治疗效果的评价。其ORR为50%，1例完全缓解，67例部分缓解，治疗的中位有效时间为49.1周。经过crizotinib治疗后，76例患者中63例（83%）肿瘤缩小，其中41例患者肿瘤缩小≥30%。就目前公布的数据，EML4-ALK阳性的NSCLC患者能从crizotinib中明显获益。

### 3.3 Ⅲ期临床试验

目前，有两项Ⅲ期临床试验正在进行。其中一项名为PROFILE1007，预计入组318例EML4-ALK阳性NSCLC患者，要求患者之前只接受过一种含铂类药物的双药化疗，将crizotinib和标准化疗药物（培美曲塞或多西紫杉醇）进行比较，主要研究终点为PFS。另一项为PROFILE1014，预计入组334例EML4-ALK阳性的NSCLC患者，将对crizotinib和培美曲塞+顺铂或培美曲塞+卡铂作为一线治疗进行对比，主要研究终点也是患者的PFS^[20]^。

## 4 EML4-ALK阳性NSCLC患者crizotinib耐药

Choi等^[21]^报道了1例EML4-ALK阳性的NSCLC患者对crizotinib产生了耐药，发现患者用药后*EML4-ALK*融合基因出现两类点突变（C1156Y和L1196M），可能与crizotinib耐药有关。Doebele等^[22]^发现14例EML4-ALK阳性的NSCLC患者服用crizotinib后疾病进展，4例出现了*ALK*二次突变，其中2例G1269A突变，另外2例中，1例*ALK*基因拷贝数增加，1例由于*EGFR*突变而无持续的*ALK*基因重排。Sasaki等^[2]^发现一例炎性成肌纤维细胞瘤患者中存在*RNA-BP2-ALK*阳性突变，这种突变降低了Ba/F3细胞对crizotinib的敏感性。目前对crizotinib的研究处于早期阶段，相关问题仍需进一步研究。

## 5 Crizotinib对ROS1阳性NSCLC有良好抗肿瘤活性

*ROS1*基因重排是新发现的NSCLC一个分子亚型，在NSCLC突变基因中占1%-2%。在体外细胞试验ROS1重排可引起癌基因*ROS1*融合激酶的表达以及增强对ROS激酶抑制剂的敏感性。*ROS1*基因突变的癌症患者常见于年龄偏小，不吸烟，病理类型为腺癌的肺癌^[7]^。

在前期临床研究中，crizotinib已显示出对抗ROS1驱动的肿瘤细胞的活性。至今已治疗13例患者^[23]^，均为通过break-apart荧光原位杂交技术（fluorescence *in situ* hybridization, FISH）筛选出的ROS1阳性的转移性NSCLC患者，给予crizotinib（250 mg, bid）。结果显示：12例患者目前仍在治疗中，对其中14例患者疗效评价，客观有效率为54%，疾病控制率为85%，治疗的中位时间为20周。不良反应与crizotinib治疗EML4-ALK阳性NSCLC患者相似。

继EML4-ALK之后，*ROS1*基因被证实为新的肺癌驱动基因，ROS1重排患者对crizotinib高度敏感，提示crizotinib可能成为ROS1重排的进展期NSCLC患者的标准治疗药物。

## 6 Crizotinib药物不良反应

在crizotinib临床试验中，29%-44%的患者因药物毒性降低使用剂量，3%-6%的患者因其而终止治疗。最常见的不良反应表现为轻度的（1/2级）视觉障碍（包括闪光幻觉、视力模糊、玻璃体飞蚊症、畏光、复视）、恶心、呕吐、腹泻、便秘、水肿。终止服药后症状可消失。另外疲劳、食欲减退、食道异常表现（如食管炎、胃食管反流等）、味觉改变、神经病变、头晕、皮疹等也有相关报道，且可耐受。最常见的严重不良反应为ALT升高和中性粒细胞减少（3/4级）。重度疲乏、呼吸困难、AST升高、血小板减少、淋巴细胞减少也有报道^[24]^。Crizotinib毒性作用较小，与传统化疗相比，患者耐受性较好。

## 7 Crizotinib治疗晚期NSCLC展望

Crizotinib是继EGFR-TKI之后NSCLC分子靶向治疗发展历程中的一个重要里程碑式的药物。从2007年发现*EML4-ALK*是NSCLC的驱动基因，到2011年crizotinib在美国上市，仅用了4年时间。美国国家综合癌症网（National Comprehensive Cancer Network, NCCN）2012年NSCLC临床指南推荐，晚期NSCLC患者在开始治疗前常规进行EML4-ALK检测，并建议阳性患者首先接受crizotinib治疗。使药物临床研发的早期就能够锁定特殊的优势人群，缩短了临床研发的宝贵时间。靶向治疗的飞速发展使人们对NSCLC患者的预后充满信心。

从目前的临床试验结果来看，crizotinib治疗EML4-ALK阳性NSCLC患者有效率高、PFS长、不良反应小。Crizotinib能否成为EML4-ALK阳性NSCLC患者的一、二线标准治疗方案之一，还有待Ⅲ期临床试验的结果以及进一步的临床研究。如何在众多NSCLC患者中筛选出*EML4-ALK*融合基因的患者，以及如何克服crizotinib耐药问题，是今后亟待解决的问题。

*ROS1*基因重排是新发现的一个NSCLC分子亚型。在前期临床研究中，crizotinib在*ROS1*基因重排NSCLC中显示了非常明显的抗肿瘤活性，具有广泛的应用前景。其缓解率、PFS、OS及安全性等还需大量的临床试验证实。越来越多的肺癌驱动基因被发现，如*K-ras*、*EGFR*、*BRAF*、*PIK3CA*、*HER-2*等基因的突变，*MET*基因扩增和*EML4-ALK*、*ROS1*基因重排。这些驱动基因的研究结果加速了NSCLC个体化治疗的进程，使更多NSCLC患者从个体化治疗中获益。
